# Genome sequence of the moderately thermophilic sulfur-reducing bacterium *Thermanaerovibrio velox* type strain (Z-9701^T^) and emended description of the genus *Thermanaerovibrio*

**DOI:** 10.4056/sigs.4237901

**Published:** 2013-10-02

**Authors:** Krishna Palaniappan, Jan P. Meier-Kolthoff, Hazuki Teshima, Matt Nolan, Alla Lapidus, Hope Tice, Tijana Glavina Del Rio, Jan-Fang Cheng, Cliff Han, Roxanne Tapia, Lynne A. Goodwin, Sam Pitluck, Konstantinos Liolios, Konstantinos Mavromatis, Ioanna Pagani, Natalia Ivanova, Natalia Mikhailova, Amrita Pati, Amy Chen, Manfred Rohde, Shanmugam Mayilraj, Stefan Spring, John C. Detter, Markus Göker, James Bristow, Jonathan A. Eisen, Victor Markowitz, Philip Hugenholtz, Nikos C. Kyrpides, Hans-Peter Klenk, Tanja Woyke

**Affiliations:** 1 DOE Joint Genome Institute, Walnut Creek, California, USA; 2 Leibniz Institute DSMZ – German Collection of Microorganisms and Cell Cultures, Braunschweig, Germany; 3 Los Alamos National Laboratory, Bioscience Division, Los Alamos, New Mexico, USA; 4 T. Dobzhansky Center for Genome Bionformatics, St. Petersburg State University, St. Petersburg, Russia; 5 Algorithmic Biology Lab, St. Petersburg Academic University, St. Petersburg, Russia; 6 Biological Data Management and Technology Center, Lawrence Berkeley National Laboratory, Berkeley, California, USA; 7 HZI – Helmholtz Centre for Infection Research, Braunschweig, Germany; 8 MTCC – Microbial Type Culture Collection & Gene Bank, CSIR-Institute of Microbial Technology, Chandigarh, India; 9 University of California Davis Genome Center, Davis, California, USA; 10 Australian Centre for Ecogenomics, School of Chemistry and Molecular Biosciences, The University of Queensland, Brisbane, Australia

**Keywords:** obligate anaerobic, motile, curved rods, organotrophic, S^0^-reduction, cyanobacterial mat, *Synergistaceae*, *Synergistetes*, GEBA

## Abstract

*Thermanaerovibrio velox* Zavarzina *et al*. 2000 is a member of the *Synergistaceae*, a family in the phylum *Synergistetes* that is already well-characterized at the genome level*.* Members of this phylum were described as Gram-negative staining anaerobic bacteria with a rod/vibrioid cell shape and possessing an atypical outer cell envelope. They inhabit a large variety of anaerobic environments including soil, oil wells, wastewater treatment plants and animal gastrointestinal tracts. They are also found to be linked to sites of human diseases such as cysts, abscesses, and areas of periodontal disease. The moderately thermophilic and organotrophic *T. velox* shares most of its morphologic and physiologic features with the closely related species, *T. acidaminovorans*. In addition to Su883^T^, the type strain of *T. acidaminovorans,* stain Z-9701^T^ is the second type strain in the genus *Thermanaerovibrio* to have its genome sequence published. Here we describe the features of this organism, together with the non-contiguous genome sequence and annotation. The 1,880,838 bp long chromosome (non-contiguous finished sequence) with its 1,751 protein-coding and 59 RNA genes is a part of the *** G****enomic* ***E****ncyclopedia of* ***Bacteria**** and* ***Archaea***** project.

## Introduction

Strain Z-9701^T^ (= DSM 12556) is the type strain of the species *Thermanaerovibrio velox* [1] in the bispecific genus *Thermanaerovibrio* [2]. The strain was isolated in 1997 from a sample of a cyanobacterial mat from the Uzon caldera in Kamchatka (Russia) [1]. The genus name is derived from the Greek words “thermos”, hot, “an”, not, and “aeros”, air, and the Neo-Latin “*vibrio*”, that vibrates, meaning a thermophilic vibrating anaerobe [2]. The species epithet is derived from the Latin adjective “*velox”*, quick, rapid [1]. In addition to the type species, *Thermanaerovibrio acidaminovorans* [2], *T. velox* is the only other member of the genus *Thermanaerovibrio* [3]. In the decade following the isolation of strain Z-9701^T^ and description of the species *T. velox*, the name was never mentioned in any abstract appearing in PubMed. Here we present a summary classification and a set of features for *T. velox* Z-9701^T^, together with the description of the genomic sequencing and annotation.

## Classification and features

A representative genomic 16S rRNA gene sequence of strain Z-9701^T^ was compared using NCBI BLAST [4,5] under default settings (e.g., considering only the high-scoring segment pairs (HSPs) from the best 250 hits) with the most recent release of the Greengenes database [6] and the relative frequencies of taxa and keywords (reduced to their stem [7]) were determined, weighted by BLAST scores. The most frequently occurring genera were *Thermanaerovibrio* (83.8%), *Aminomonas* (8.5%) and *Thermovirga* (7.7%) (9 hits in total). Regarding the two hits to sequences from members of the species, the average identity within HSPs was 96.7%, whereas the average coverage by HSPs was 100.5%. Regarding the four hits to sequences from other members of the genus, the average identity within HSPs was 94.9%, whereas the average coverage by HSPs was 96.4%. Among all other species, the one yielding the highest score was *T. acidaminovorans* (CP001818), which corresponded to an identity of 95.3% and an HSP coverage of 99.7%. (Note that the Greengenes database uses the INSDC (= EMBL/NCBI/DDBJ) annotation, which is not an authoritative source for nomenclature or classification.) The highest-scoring environmental sequence was AF280820 ('bioreactor clone tbr1-2'), which showed an identity of 94.7% and an HSP coverage of 99.7%. The most frequently occurring keywords within the labels of all environmental samples which yielded hits were 'digest' (12.2%), 'anaerob' (7.2%), 'wastewat' (6.6%), 'mesophil' (6.5%) and 'treat' (6.4%) (241 hits in total), indicating that close relatives of *T. velox* could also thrive at lower temperatures in anaerobic aqueous environments*.* Environmental samples which yielded hits of a higher score than the highest scoring species were not found.

**Figure 1:**
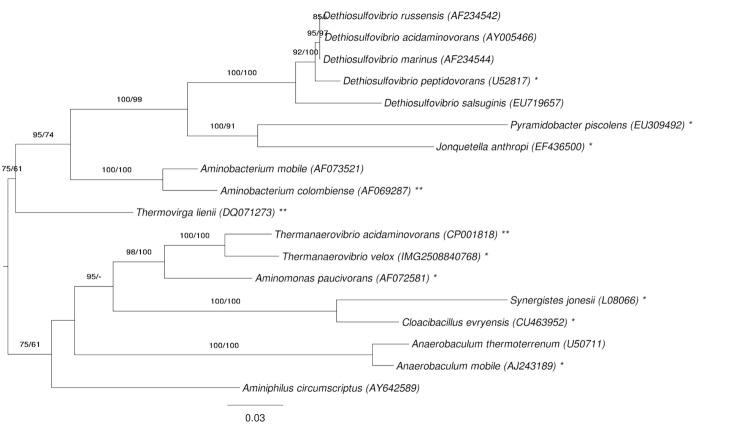
Phylogenetic tree highlighting the position of *T.velox* relative to the type strains of the other species within the phylum *'Synergistetes'*. The tree was inferred from 1,348 aligned characters [8,9] of the 16S rRNA gene sequence under the maximum likelihood (ML) criterion [10]. Rooting was done initially using the midpoint method [11] and then checked for its agreement with the current classification (Table 1). The branches are scaled in terms of the expected number of substitutions per site. Numbers adjacent to the branches are support values from 150 ML bootstrap replicates [12] (left) and from 1,000 maximum-parsimony bootstrap replicates [13] (right) if larger than 60%. Lineages with type strain genome sequencing projects registered in GOLD [14] are labeled with one asterisk, those also listed as 'Complete and Published' with two asterisks [15-17]. *Aminomonas paucivorans* [18] and *Dethiosulfovibrio peptidovorans* [19] lack the second asterisks because these are permanent draft genome sequences (for *Jonquetella anthropi* see AGRU00000000).

Figure 1 shows the phylogenetic neighborhood of *T. velox* in a 16S rRNA based tree. The sequences of the three 16S rRNA gene copies in the genome differ from each other by up to one nucleotide, and differ by up to 45 nucleotides from the previously published 16S rRNA gene sequence (AF161069), which contains 38 ambiguous base calls. This sequence was recently updated by (FR733707) of the SOS initiative [26], which perfectly matches the 16S rRNA gene copies in the genome.

**Table 1:** Classification and general features of *T. velox* Z9701^T^ according to the MIGS recommendations [20] (published by the Genome Standards Consortium [21]) and the NamesforLife database [3].

**MIGS ID**	**Property**	**Term**	**Evidence code**
	Current classification	Domain *Bacteria*	TAS [22]
		Phylum ‘*Synergistetes’*	TAS [23]
		Class *Synergistia*	TAS [23]
		Order *Synergistales*	TAS [23]
		Family *Synergistaceae*	TAS [23]
		Genus *Thermanaerovibrio*	TAS [1,2]
		Species *Thermanaerovibrio velox*	TAS [1]
		Type strain Z-9701	TAS [1]
	Gram stain	negative	TAS [1]
	Cell shape	curved rods	TAS [1]
	Motility	motile	TAS [1]
	Sporulation	non-sporulating	
	Temperature range	thermophile, 45-70°C	TAS [1]
	Optimum temperature	60-65°C	TAS [1]
	Salinity	no NaCl required for growth, but can tolerate up to 35 g l^—1^	TAS [1]
MIGS-22	Oxygen requirement	obligate anaerobe	TAS [1]
	Carbon source	glucose, fructose, mannose, yeast extract	TAS [1]
	Energy metabolism	organotrophic sulfur-reducer	TAS [1]
MIGS-6	Habitat	hot spring	TAS [1]
MIGS-15	Biotic relationship	free living	TAS [1]
MIGS-14	Pathogenicity	none	NAS
	Biosafety level	1	TAS [24]
MIGS-23.1	Isolation	cyanobacterial mat	TAS [1]
MIGS-4	Geographic location	Uzon caldera, Kamchatka, Russia	TAS [1]
MIGS-5	Sample collection time	1997 or earlier	NAS
MIGS-4.1 MIGS-4.2	Latitude – Longitude	54.519 – 159.976	NAS
MIGS-4.3	Depth	not reported	
MIGS-4.4	Altitude	about 1617 m	NAS

Evidence codes - TAS: Traceable Author Statement; NAS: Non-traceable Author Statement, based on a generally accepted property for the species, or anecdotal evidence. Evidence codes are from of the Gene Ontology project [25].

**Figure 2:**
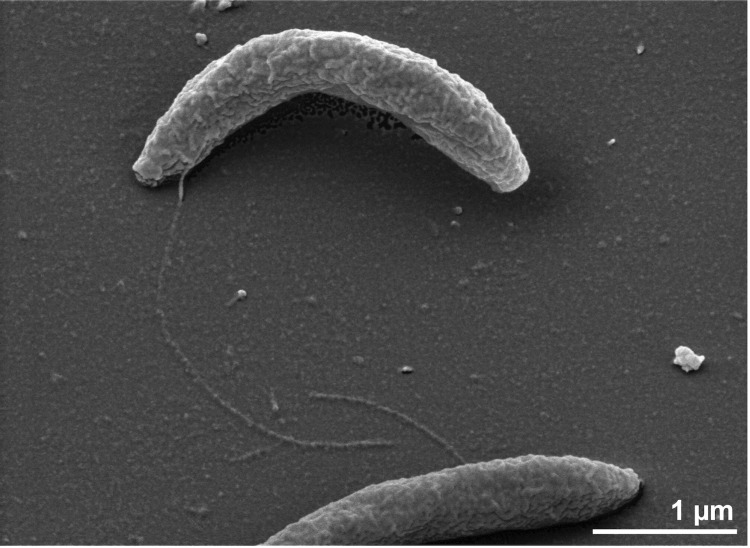
Scanning electron micrograph of *T. velox* Z9701^T^

Cells of strain Z-9701^T^ are curved rods, 0.5-0.7 × 2.5-5.0 µm in size (Figure 2) [1], stain Gram-negative and are motile *via* lateral flagella located on the concave side. Colonies are 0.2 mm wide, round and irregular with even edges [1], growing strictly anaerobically at optima of 60-65°C and pH 7.3 while fermenting a variety of sugars, but also when grown on yeast extract and Casamino acids [1]. Acetate, lactate, H_2_, CO_2_ and ethanol are the fermentation products formed during growth on glucose [1]. During organotrophic growth on glucose or peptides strain Z-9701^T^ reduces elemental sulfur to H_2_S [1]. The strain is also capable of lithotrophic growth in the presence of elemental sulfur with molecular hydrogen as the energy source and yeast extract as the carbon source [1].

### Chemotaxonomy

No chemotaxonomical data reported so far. The G+C content was reported as only 54.6 mol% based on thermal denaturation [1], 4.2% below the value determined by genome sequencing (see below).

## Genome sequencing and annotation

### Genome project history

This organism was selected for sequencing on the basis of its phylogenetic position [27], and is part of the *** G****enomic* ***E****ncyclopedia of* ***Bacteria**** and* ***Archaea***** project [28]. The genome project is deposited in the Genomes On Line Database [14] and the complete genome sequence is deposited in GenBank. Sequencing, finishing and annotation were performed by the DOE Joint Genome Institute (JGI) using state of the art sequencing technology [29]. A summary of the project information is shown in Table 2.

**Table 2:** Genome sequencing project information

**MIGS ID**	**Property**	**Term**
MIGS-31	Finishing quality	Non-contiguous
MIGS-28	Libraries used	Three genomic libraries: one 454 pyrosequence standard library, one 454 PE library (7 kb insert size), one Illumina library
MIGS-29	Sequencing platforms	Illumina GAii, 454 GS FLX Titanium
MIGS-31.2	Sequencing coverage	120.0 × Illumina; 7.9 × pyrosequence
MIGS-30	Assemblers	Newbler version 2.3, Velvet 1.0.13, phrap version SPS - 4.24
MIGS-32	Gene calling method	Prodigal
	INSDC ID	CM001377, AHGV00000000
	GenBank Date of Release	December 19, 2011
	GOLD ID	Gi05367
	NCBI project ID	65505
	Database: IMG	2508501068
MIGS-13	Source material identifier	DSM 12556
	Project relevance	Tree of Life, GEBA

### Growth conditions and DNA isolation

*T. velox* strain Z-9701^T^, DSM 12556, was grown anaerobically (with 8:2 N_2_/CO_2_ v/v in the head space) in DSMZ medium 873 (*Thermanaerovibrio* medium) [30] at 60°C. DNA was isolated from 0.5-1 g of cell paste using Jetflex Genomic DNA Purification kit (GENOMED 600100) following the standard protocol as recommended by the manufacturer, but with an additional step for improved cell lysis: 30 min incubation with additional 40 µl protease K at 58°C. DNA is available through the DNA Bank Network [31].

### Genome sequencing and assembly

The genome was sequenced using a combination of Illumina and 454 sequencing platforms. All general aspects of library construction and sequencing can be found at the JGI website [32]. Pyrosequencing reads were assembled using the Newbler assembler (Roche). The initial Newbler assembly consisting of 32 contigs in one scaffold was converted into a phrap [33] assembly by making fake reads from the consensus, to collect the read pairs in the 454 paired end library. Illumina GAii sequencing data (5,956 Mb) was assembled with Velvet [34] and the consensus sequences were shredded into 1.5 kb overlapped fake reads and assembled together with the 454 data. The 454 draft assembly was based on 29.5Mb 454 draft data and all of the 454 paired end data. Newbler parameters are -consed -a 50 -l 350 -g -m -ml 20. The Phred/Phrap/Consed software package [33] was used for sequence assembly and quality assessment in the subsequent finishing process. After the shotgun stage, reads were assembled with parallel phrap (High Performance Software, LLC). Possible mis-assemblies were corrected with gapResolution [32], Dupfinisher [35], or sequencing cloned bridging PCR fragments with subcloning. Gaps between contigs were closed by editing in Consed, by PCR and by Bubble PCR primer walks (J.-F. Chang, unpublished). A total of 46 additional reactions and one shatter library were necessary to close gaps and to raise the quality of the final sequence. Illumina reads were also used to correct potential base errors and increase consensus quality using the software *Polisher* developed at JGI [36]. The error rate of the final genome sequence is less than 1 in 100,000. Together, the combination of the Illumina and 454 sequencing platforms provided 127.9 × coverage of the genome. The final assembly contained 102,371 pyrosequence and 3,000,000 Illumina reads.

### Genome annotation

Genes were identified using Prodigal [37] as part of the DOE-JGI [38] genome annotation pipeline, followed by a round of manual curation using the JGI GenePRIMP pipeline [39]. The predicted CDSs were translated and used to search the National Center for Biotechnology Information (NCBI) non-redundant database, UniProt, TIGRFam, Pfam, PRIAM, KEGG, COG, and InterPro databases. These data sources were combined to assert a product description for each predicted protein. Additional gene prediction analysis and functional annotation was performed within the Integrated Microbial Genomes - Expert Review (IMG-ER) platform [40].

## Genome properties

**Figure 3:**
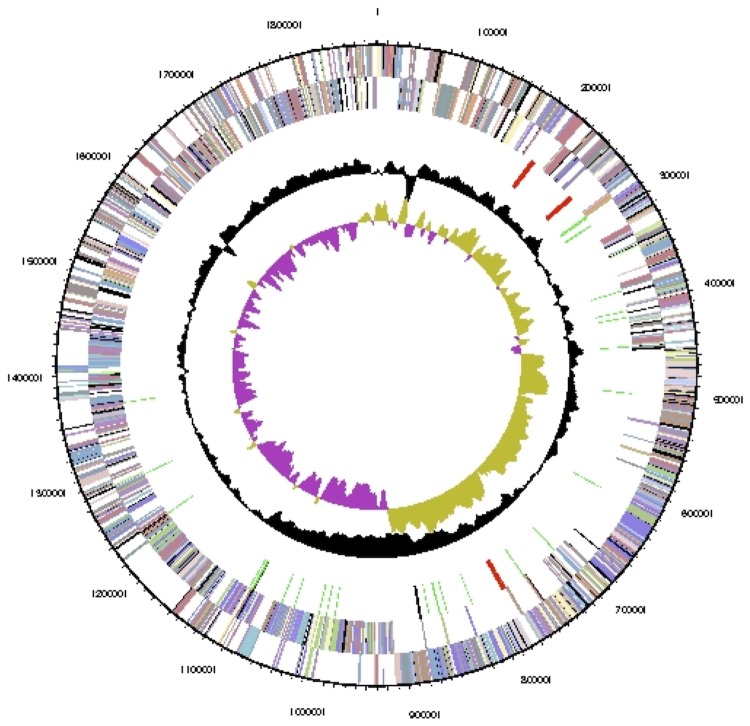
Graphical map of the chromosome. From outside to the center: Genes on forward strand (colored by COG categories), Genes on reverse strand (colored by COG categories), RNA genes (tRNAs green, rRNAs red, other RNAs black), GC content(black), GC skew (purple/olive).

The draft genome consist of one circular chromosome of 1,880,838 bp length with a 58.8% G+C content (Table 3 and Figure 3). Of the 1,810 genes predicted, 1,751 were protein-coding genes, and 59 RNAs; 12 pseudogenes were also identified. The majority of the protein-coding genes (84.4%) were assigned a putative function while the remaining ones were annotated as hypothetical proteins. The distribution of genes into COGs functional categories is presented in Table 4.

**Table 3:** Genome Statistics

**Attribute**	Value	% of Total
Genome size (bp)	1,880,838	100.00%
DNA coding region (bp)	1,724,402	91.68%
DNA G+C content (bp)	1,110,498	58.78%
Number of replicons	1	
Extrachromosomal elements	0	
Total genes	1,810	100.00%
RNA genes	59	3.26%
rRNA operons	3	
tRNA genes	48	2.65%
Protein-coding genes	1,751	96.74%
Pseudo genes	12	0.66%
Genes with function prediction (proteins)	1,527	84.36%
Genes in paralog clusters	617	34.09%
Genes assigned to COGs	1,539	85.03%
Genes assigned Pfam domains	1,524	84.20%
Genes with signal peptides	285	15.75%
Genes with transmembrane helices	407	22.49%
CRISPR repeats	1	

**Table 4:** Number of genes associated with the general COG functional categories

**Code**	**Value**	**%age**	**Description**
J	150	8.8	Translation, ribosomal structure and biogenesis
A	0	0.0	RNA processing and modification
K	83	4.9	Transcription
L	70	4.1	Replication, recombination and repair
B	0	0.0	Chromatin structure and dynamics
D	28	1.7	Cell cycle control, cell division, chromosome partitioning
Y	0	0.0	Nuclear structure
V	17	1.0	Defense mechanisms
T	105	6.2	Signal transduction mechanisms
M	102	6.0	Cell wall/membrane biogenesis
N	74	4.4	Cell motility
Z	0	0.0	Cytoskeleton
W	0	0.0	Extracellular structures
U	39	2.3	Intracellular trafficking and secretion, and vesicular transport
O	55	3.2	Posttranslational modification, protein turnover, chaperones
C	128	7.5	Energy production and conversion
G	96	5.7	Carbohydrate transport and metabolism
E	200	11.8	Amino acid transport and metabolism
F	63	3.7	Nucleotide transport and metabolism
H	99	5.8	Coenzyme transport and metabolism
I	34	2.0	Lipid transport and metabolism
P	63	3.7	Inorganic ion transport and metabolism
Q	22	1.3	Secondary metabolites biosynthesis, transport and catabolism
R	154	9.1	General function prediction only
S	116	6.8	Function unknown
-	271	15.0	Not in COGs

## Insights into the genome sequence

### Comparative genomics

The phylum *Synergistetes* is one of the more recently proposed phyla in the domain *Bacteria*, posited only four years ago by Jumas-Bilak *et al*. [23]. As of today the phylum contains only one order, *Synergistales*, with one family, *Synergistaceae*, including 11 genera with 18 species (see Figure 1). The members of the phylum are extremely well characterized on the genomic level, with 12 out of the 18 type strains for the member species having already completed or ongoing genome sequencing projects, one type strain targeted for sequencing (*Anaerobacterium thermoterrum*) and only four type strains currently not indicated for genome sequencing in the Genomes On Line Database (GOLD) [14]. Here we present a brief comparison of the genome of *T. velox* with its closest phylogenetic neighbors (according to Figure 1): *T. acidamonovorans* [17] and *A. paucivorans* [18].

The genomes of the two recently sequenced *Thermanaerovibrio* type strains differ only slightly in their size, *T. velox* having 1.88 Mbp and *T. acidaminovorans* 1.84 Mbp and their total number of genes, 1,810 and 1,825, respectively. *A. paucivorans*, on the other hand, has a significantly larger genome with 2,494 genes on 2.63 Mbp. An estimate of the overall similarity between *T. velox* with both, *T. acidaminovorans* and *A. paucivorans,* was generated with the GGDC-Genome-to-Genome Distance Calculator [41-43]. This system calculates the distances by comparing the genomes to obtain HSPs (high-scoring segment pairs) and inferring distances from the set of formulas (1, HSP length / total length; 2, identities / HSP length; 3, identities / total length). For convenience, the GGDC also reports model-based DDH estimates along with their confidence intervals [21,41]. Table 5 shows the results of the pairwise comparison.

**Table 5:** Pairwise comparison of *T. velox* with *T. acidaminovorans* and *A. paucivorans* using the GGDC-Genome-to-Genome Distance Calculator.

		**HSP length /** **total length [%]**	**Identities /** **HSP length [%]**	**Identities /** **total length [%]**
*T. velox*	*T. acidaminovorans*	44	78	35
*T. velox*	*A. paucivorans*	8	78	7
*T. acidaminovorans*	*A. paucivorans*	17	77	13

The comparison of *T. velox* with *T. acidaminovorans* reached the highest scores using the GGDC, 44% of the average of genome length are covered with HSPs (Table 5). The identity within the HSPs was 78%, whereas the identity over the whole genome was 35%. Lower similarity scores were observed in the comparison of *T. velox* with *A. paucivorans,* only 17% of the average of both genome lengths are covered with HSPs. The identity within these HSPs was 77%, whereas the identity over the whole genome was only 13%.

With regard to *T. velox* and *T. acidaminovorans* the corresponding DDH estimates were below the 70% threshold under formulas 1-3 throughout: 27.2% (±3.5), 20.4% (±2.3) and 24.6% (±3.0). The DDH estimated confidence intervals are given in parentheses as provided by [41]. These results are in line with a previously reported wet-lab DDH value of 15% (±1) [1]

As expected, those distances relating HSP coverage (formula 1) and number of identical base pairs within HSPs to total genome length (formula 3) are higher between the *T. velox* and *T. acidaminovorans* than between *T. velox* and *A. paucivorans*. That the distances relating the number of identical base pairs to total HSP length (formula 2) behave differently indicates that the genomic similarities between *T. velox, T. acidaminovorans* and *A. paucivorans* are strongly restricted to more conserved sequences, a kind of saturation phenomenon [42].

In order to compare the *T. velox* and *T. acidaminovorans* genomes, correlation values (Pearson coefficient) according to the similarity on the level of COG category, pfam and TIGRfam were calculated. A very high correlation value (0.98) was reached on the level of pfam data; the correlation values on the basis of COG and TIGRfam data were only slightly smaller; 0.95 and 0.97, respectively. As a correlation value of 1 indicates the highest correlation, we can find a near perfect correlation between the genomes of *T. velox* and *T. acidaminovorans* considering the above data [40].

The comparison of the number of genes belonging to different COG categories revealed no large differences in the genomes of *T. velox* and *T. acidaminovorans*, with only 0.2% deviation between the same COG categories on average. A slightly higher fraction of genes belonging to the categories amino acid metabolism (*T. velox* 11.8%, *T. acidaminovorans* 11.4%), carbohydrate metabolism (*T. velox* 5.7%, *T. acidaminovorans* 5.3%) and defense mechanisms (*T. velox* 1.0%, *T. acidaminovorans* 0.7%) were identified in *T. velox*. The gene count in further COG categories such as cell cycle control, cell motility, cell biogenesis, lipid metabolism, secondary catabolism, *posttranslational modification* and signal transduction was also slightly increased in *T. velox*, *but differed at most by 5 genes*. In contrast, a slightly smaller fraction of genes belonging to the categories translation (*T. velox* 8.8%, *T. acidaminovorans* 9.2%), nucleotide metabolism (*T. velox* 3.7%, *T. acidaminovorans* 4.0%), transcription (*T. velox* 4.9%, *T. acidaminovorans* 5.1%), replication systems (*T. velox* 4.1%, *T. acidaminovorans* 4.3%) and inorganic ion transport and metabolism (*T. velox* 3.7%, *T. acidaminovorans* 3.9%) were also identified in *T. velox*. The remaining COG categories of intracellular transport, energy production/conversion and coenzyme metabolism differed at most by two genes.

**Figure 4:**
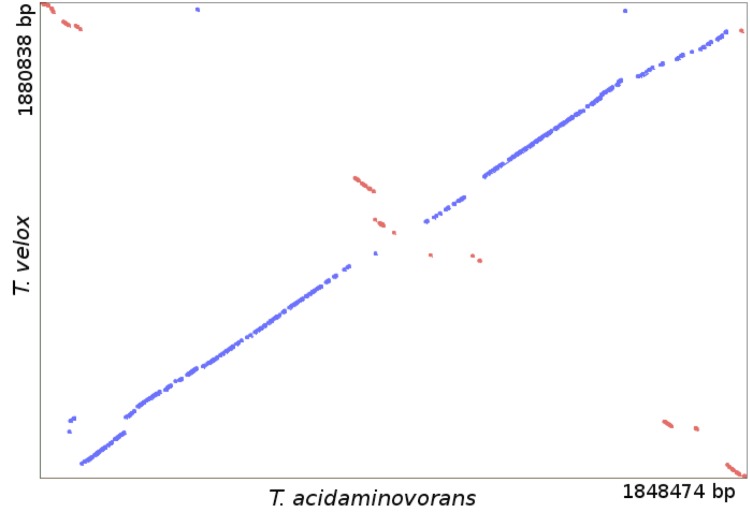
Synteny dot blot based on the genome sequences of *T. velox* and *T. acidaminovorans*. Blue dots represent regions of similarity found on parallel strands, and red dots show regions of similarity found on anti-parallel strands.

The synteny dot plot in Figure 4 shows a nucleotide-based comparison of the two *Thermanaerovibrio* genomes. In most parts of the genomes, a high degree of similarity becomes visible with only a small number of indels. There exists a pronounced collinearity between the two genomes.

**Figure 5:**
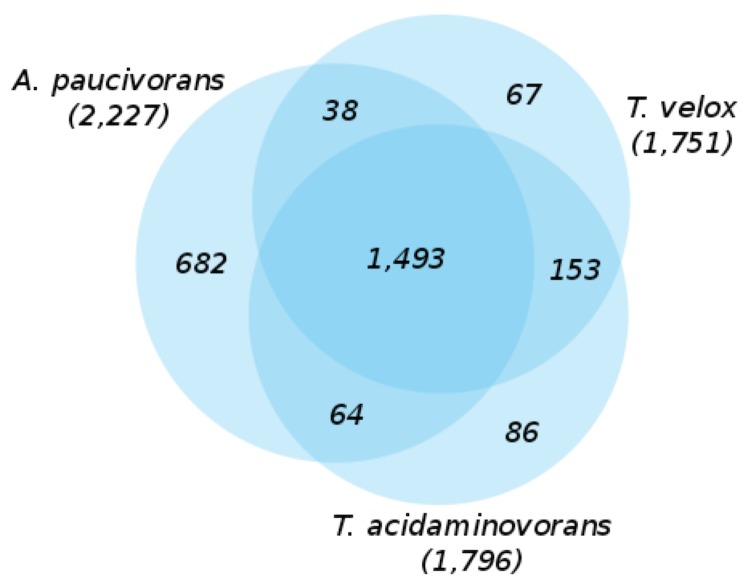
Venn-diagram depicting the intersections of protein sets (total numbers in parentheses) of *T. velox*, *T. acidaminovorans* and *A. paucivorans*.

The Venn-diagram (Figure 5) shows the number of shared genes in the genomes of the three closely related type strains. *T. velox* and *T. acidaminovorans* share a significant number of 153 genes that are not present in the genome of *A. paucivorans* [18]. A huge fraction of these genes are involved in transport functions, such as genes coding for TRAP-type C4-dicarboxylate transport systems, ABC-type dipeptide transport systems, ABC-type dipeptide/oligopeptide/nickel transport systems, ABC-type hemin transport systems, p-aminobenzoyl-glutamate transporters, Na+/H+-dicarboxylate symporters, sugar phosphate permeases, ABC-type Fe3+ transport systems, fructose-specific PTS systems, glucose-specific PTS systems, molybdenum ABC transporters, Na+/H+-dicarboxylate symporters, biopolymer transport proteins, Mg2+ transporters, Na+/H+ antiporters, NhaD and related arsenite permeases, sodium--glutamate symport carrier and xanthine permeases. But also genes for transcriptional regulators of sugar metabolism, peptidase T-like protein, sugar transferases involved in lipopolysaccharide synthesis, L-aspartate oxidase, quinolinate synthetase complex, DNA modification/repair radical SAM protein, glycosyltransferase family 10 (fucosyltransferase), phosphoheptose isomerase, phosphomannose isomerase, phosphoribosyl-dephospho-CoA transferase (holo-ACP synthetase), methyl-accepting chemotaxis protein, and ethanolamine utilization protein.

The significant difference between the previously reported G+C content of strain Z-9701^T^, 54.6% [1] and the G+C content as inferred from the draft genome sequence, 58.8% (Table 3), as well as the similarly significant difference between the G+C content reported for the type strain of the other validly named species in the genus, *T. acidaminovorans* [2], Su883^T^, 56.6% [2] vs. 63.8% from the genome sequence [17] demands the emendation of the species and genus descriptions, which were last updated by Baena *et al.* 1999 [2] and Zavarzina *et al.* in 2000 [1].

### Emended description of the species ***Thermanaerovibrio acetaminovorans*** Guangsheng ***et al.*** 1997 emend. Baena ***et al.*** 1999

The description of the species *Thermanaerovibrio acetaminovorans* is the one given by Baena *et al*. [2], with the following modification. The G+C content is 63.8 mol% [17].

### Emended description of the species ***Thermanaerovibrio velox*** Zavarzina ***et al.*** 2000

The description of the species *Thermanaerovibrio velox* is the one given by Zavarzina *et al*. [1], with the following modification. The G+C content is 58.8 mol% .

### Emended description of the genus ***Thermanaerovibrio*** Baena ***et al.*** 1999 emend. Zavarzina ***et al***. 2000 

The description of the genus *Thermanaerovibrio* is the one given by Zavarzina *et al*. [1], with the following modification. The G+C content is between 58.8 and 63.8 mol%.
